# Genomic content typifying a prevalent clade of bovine mastitis-associated *Escherichia coli*

**DOI:** 10.1038/srep30115

**Published:** 2016-07-20

**Authors:** Robert J. Goldstone, Susan Harris, David G. E. Smith

**Affiliations:** 1 Heriot-Watt University, School of Life Sciences, Edinburgh Campus, EH14 4AS, Scotland.

## Abstract

*E. coli* represents a heterogeneous population with capabilities to cause disease in several anatomical sites. Among sites that can be colonised is the bovine mammary gland (udder) and a distinct class of mammary pathogenic *E. coli* (MPEC) has been proposed. MPEC are the principle causative agents of bovine mastitis in well-managed dairy farms, costing producers in the European Union an estimated €2 billion per year. Despite the economic impact, and the threat this disease presents to small and medium sized dairy farmers, the factors which mediate the ability for *E. coli* to thrive in bovine mammary tissue remain poorly elucidated. Strains belonging to *E. coli* phylogroup A are most frequently isolated from mastitis. In this paper, we apply a population level genomic analysis to this group of *E. coli* to uncover genomic signatures of mammary infectivity. Through a robust statistical analysis, we show that not all strains of *E. coli* are equally likely to cause mastitis, and those that do possess specific gene content that may promote their adaptation and survival in the bovine udder. Through a pan-genomic analysis, we identify just three genetic loci which are ubiquitous in MPEC, but appear dispensable for *E. coli* from other niches.

*Escherichia coli* is a diverse group of Gram-negative bacteria that can colonise and exploit a range of environments and hosts^1^. These bacteria often asymptomatically colonise the digestive tract of mammals, although various *E. coli* types can also cause severe gastrointestinal disease and a range of extra-intestinal infections in both humans and animals^2^,^3^. Phylogenetically, *E. coli* can be subdivided into several groups which have been termed phylogroups A, B1, B2, C, D, E, and F^4^. In addition to these phylogroups, dispersed within the population structure of *E. coli*, are strains designated as *Shigella* that are of polyphyletic origin^5^,^6^.

Although *E. coli* from phylogroups A and B1 are generally considered to be less pathogenic and more likely to be commensals than isolates from other phylogroups^3^,^7^, these phylogroups do include significant pathogens^8^,^9^. Indeed, several studies have shown that the vast majority of *E. coli* isolates from cases of bovine mastitis (termed mammary pathogenic *E. coli*, or MPEC) originate from within these two phylogroups^10^,^11^,^12^,^13^,^14^.

Some lines of evidence suggest that mastitis is a general reaction to contamination of the bovine udder with any *E. coli* strain. For instance, the inflammatory symptoms of mastitis can be experimentally elicited by intra-mammary infusion of lipopolysaccharide (LPS) derived from cultures of *E. coli* of various serotypes^15^,^16^,^17^. Furthermore, some research suggests that the severity of disease is more closely dependent on factors related to the bovine host than any intrinsic differences between bacterial strains^18^. Previous investigations of MPEC virulence factor carriage using PCR-based screens have also been broadly unsuccessful in identifying, or agreeing upon, a core set of factors which are associated with MPEC^10^,^13^,^14^,^19^,^20^. However, recent evidence suggests that mastitis-causing capability in *E. coli* is not a general ability – for example, Blum *et al*.^21^ showed that an environmental isolate termed *E. coli* K71 was incapable of causing experimental mastitis in either mice or cows^21^. This, along with the evidence that the molecular diversity of mastitis isolates compared with other *E. coli* is limited^10^,^22^, supports the conjecture that the bovine udder environment presents a milieu which is selective against the successful colonisation by some *E. coli* strains, yet is permissive for others.

Recently, there has been a small number of publications which have begun to examine MPEC cohorts at the genomic level^21^,^23^,^24^,^25^,^26^ however, few studies have examined MPEC genomes in any detail^21^,^23^. For example, Richards *et al*.^23^ compared the genome sequences of four MPEC isolates with eleven genomes of reference ‘commensal’ strains, such as MG1655 and HS, and principally identified a type six secretion system (T6SS), conserved in MPEC yet only sporadically present in commensal strains^23^. Blum *et al*.^21^ analysed the genome sequences of three MPEC isolates in comparison with the avirulent strain K71^21^. In that study, the authors identified a complement of 197 genes which were present in the genomes of MPEC, yet absent in K71. These genes included those involved in the synthesis of LPS and other membrane antigens, the capture of iron from ferric citrate, and the metabolism of certain sugars^21^. A recent study by Kempf *et al*.^26^ with five MPEC isolates also struggled to make progress in identifying genes implicated in the MPEC phenotype^26^. This study identified fifty-nine gene families which the authors postulate are MPEC-specific, however the use of somewhat relaxed inclusion criteria (presence in only two of five MPEC isolates) may reduce the likelihood that these genes impact on the MPEC phenotype. That study then used a classical candidate gene approach where they highlighted the possible role for systems such as iron acquisition, fimbriae and LPS^26^. All of these analyses are subject to small sample size limitations, which was recognised by Richards *et al*.^23^, and there is little overlap in the genes identified as putative MPEC factors. Given the lack of definitive genes implicated in the MPEC phenotype by these studies, a systematic evaluation is necessary.

In the present study, we use a comprehensive population-level genomics approach to analyse sixty-six MPEC isolates. Since phylogroup A strains appear enriched within MPEC isolates when compared with their environmental abundance^10^ and their frequency in bovine faeces^27^,^28^ , this is indicative of an active selective process which enriches phylogroup A organisms in this niche. Also, since phylogroup A *E. coli* are often found to be the principal *E. coli* clade implicated in mastitis, this led us to focus the present study exclusively on MPEC originating from within this group.

In this paper we present strong evidence that not all *E. coli* from phylogroup A are equally likely to be capable of causing mastitis, and uncover a specific set of just three genetic loci which appear to constitute major genomic determinants specifying phylogroup A MPEC. Our evidence suggests that MPEC originate within strongly-selected lineages within phylogroup A and, as a population, these MPEC are significantly more closely-related to each other than would be expected from a random distribution of these isolates across the phylogroup A population structure. Furthermore, this restriction in molecular diversity observed in MPEC is mirrored by a more limited pan-genome and an expanded core genome repertoire in this population, compared with what may be expected from phylogroup A *E. coli* in general. These observations are consistent with the hypothesis that a selective process results in a specific sub-population of MPEC recruited from the wider phylogroup A population. Lastly, to identify candidate genes which are associated with the MPEC lifestyle, yet dispensable for other *E. coli*, we searched for genes which were present in the core genome of MPEC, but did not tend to be represented in the core genome of comparatively sized random samples of the wider phylogroup A population. This analysis resulted in the identification of nineteen genes which cluster into only three genetic loci. These loci, which include the *ycdU-ymdE* genes, the phenylacetic acid degradation pathway and the ferric citrate uptake system, are strong candidates for genes mediating the ability for phylogroup A *E. coli* to survive and thrive in the bovine udder.

## Results and Discussion

**Figure 1:**
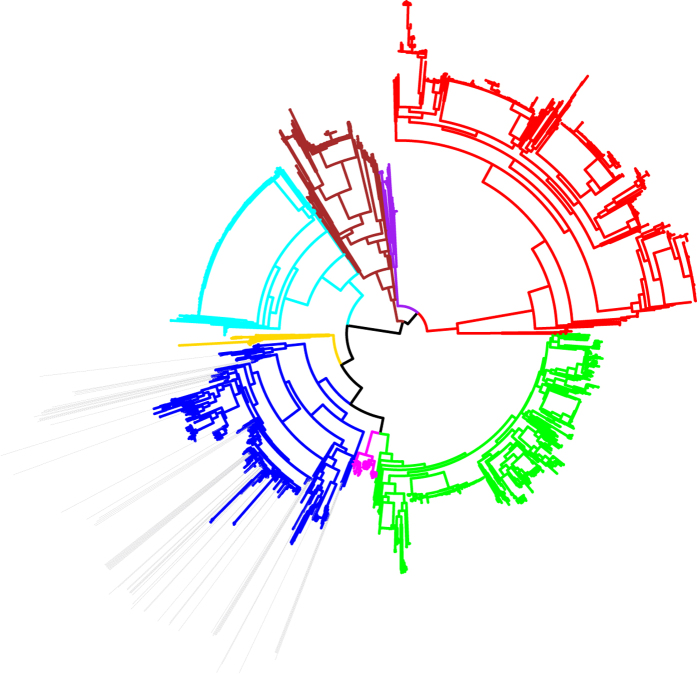
Position of 66 mastitis-associated *E. coli* isolates within phylogroup A. A maximum likelihood tree constructed from the concatenated sequence of 159 core *E. coli* genes elaborates the known population structure of *E. coli*. Using this tree, we positioned the 66 MPEC isolate within phylogroup A (grey bars). Branches are coloured according to phylogroup: (**A**) blue; (**B1**), green; (**B2**), red; (**C**) magenta; (**D**) brown; (**E**) cyan; F purple; *Shigella*; gold. *Shigella* genomes which fall into other phylogroups are not coloured.

In order to capture the breadth of the population of phylogroup A *E. coli* in mastitis, we confirmed the position of sixty-two newly sequenced *E. coli* MPEC, isolated from several countries, into phylogroup A. Four previously published MPEC genome sequences from NCBI (P4, 1303, ECC-Z, D6-117_07.11) also originate within phylogroup A, and these were included in our analyses. Figure 1 shows the positioning of the final panel of these sixty-six phylogroup A MPEC genome sequences within the population structure of *E. coli*. The general structure of the tree shown in Fig. 1 is highly similar to phylogenetic analyses conducted elsewhere^29^, and reflects the monophyly of the seven recognised *E. coli* phylogroups: A (blue), B1 (green), B2 (red), C (magenta), D (brown), E (cyan) and F (purple). *Shigella* (gold) are known to be of polyphyletic origin^5^,^6^ and for clarity of presentation *Shigella* embedded within other phylogroups are not individually coloured.

### Phylogroup A MPEC are more similar to each other than expected by chance

**Figure 2:**
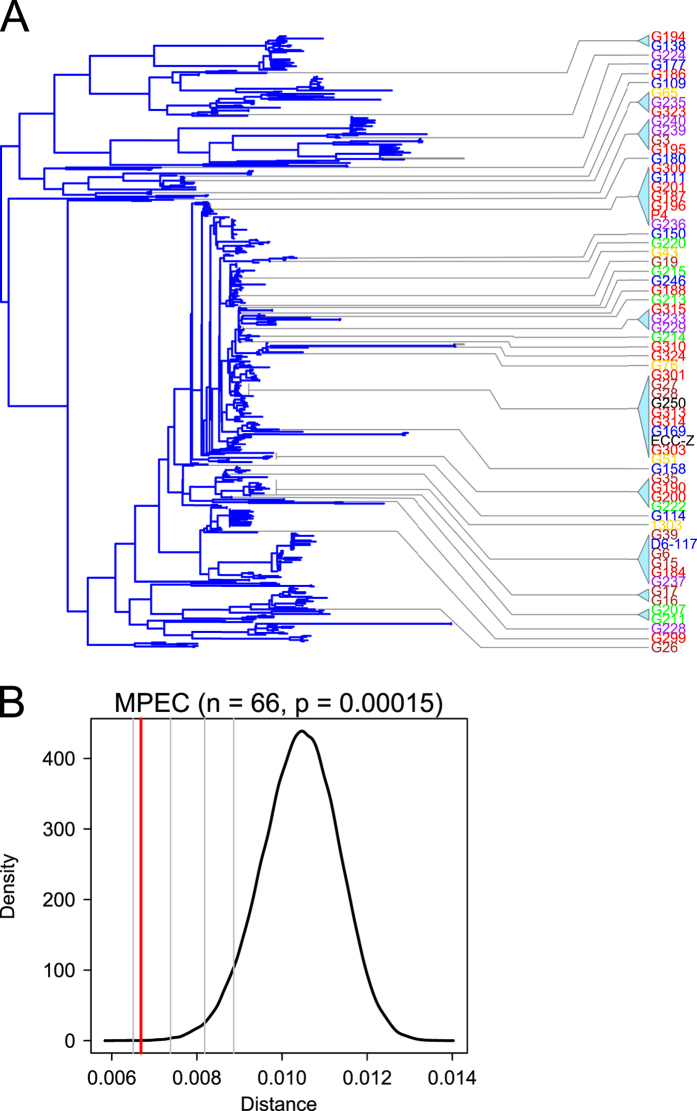
Mastitis isolates are more closely related to each other, on average, than would be expected by chance. Panel A shows a phylogenetic tree for 533 phylogroup A genomes, constructed from the concatenated sequence of 520 non-recombining genes as estimated by maximum likelihood. For clarity, bootstrap values have been removed. Labels are coloured according to country of origin (Belgium = brown, Finland = green, France = blue, Germany = purple, Israel = gold, UK = red). One isolate (ECC-Z) was isolated from the Netherlands, and one was isolated in Denmark. Panel B shows the results of a resampling analysis to investigate the probability that the average phylogenetic distance between MPEC could be generated by randomly placing MPEC genomes onto the phylogroup A phylogenetic tree. The bell curve in the plot represents the kernel density estimate of 100,000 replications, where the average distance between 66 randomly selected genomes is calculated. The red vertical line represents the actual average distance observed between MPEC. The *p*-value is calculated by how many of the randomised samples display a distance as low as, or lower, than that observed between MPEC. The distance between MPEC genomes is highly significant (p = 0.00015), indicating that only 15 in 100,000 randomised replications had average distances which were as low or lower than that observed between MPEC genomes. The four vertical grey bars represent the location on the distribution that would yield p-values of 0.0001, 0.001, 0.01, and 0.05, respectively.

Although the data in Fig. 1 shows that MPEC originate from a wide spectrum of lineages within phylogroup A, we noted that many strains appeared clustered onto closely neighbouring branches. Our hypothesis is that only certain strains of *E. coli* are capable of eliciting bovine mastitis. We reasoned we could test this hypothesis by asking whether all lineages of phylogroup A *E. coli* were equally likely to be observed in cases of mastitis. To do this, we elaborated a much more comprehensive maximum likelihood phylogenetic tree for the 533 phylogroup A isolates, using the nucleotide sequence of 520 non-recombining core phylogroup A genes, and investigated the positions of the MPEC genomes within this tree (Fig. 2A). For clarity of presentation, bootstrap values have been removed. A tree with bootstrap values is included as Additional Figure S1. This refined tree reflected our earlier observation that many MPEC have close neighbours within the tree that are also MPEC, as well as revealing that many MPEC are not epidemiologically related by country. To investigate this statistically, we reasoned that any such distinction between MPEC and the wider phylogroup A population should be reflected in the tendency for MPEC isolates to be more closely related to each other than would a random selection of phylogroup A *E. coli*. To test this, we sampled sixty-six random phylogroup A genomes from the population over 100,000 replications and, for each sample, calculated the average phylogenetic distance observed between the genomes within the sample. This distribution of average distances is shown as a density plot in Fig. 2(B). Next, we compared the actual average distance observed between the MPEC isolates with this null distribution (shown as a red vertical line in Fig. 2B), and calculated the *p* value of how likely this distance is to have been caused by chance alone by using the number of randomised samples which exhibited average distance as small or smaller than that observed between MPEC divided by the number of replications. These data show that it is incredibly unlikely (*p* = 0.00015) that an average distance as small as that observed between MPEC could be generated by random positioning of MPEC within phylogroup A. Overall, these analyses point to significant reduction in phylogenetic diversity of MPEC strains within phylogroup A compared with what may be expected if these strains were randomly positioned within the phylogroup A population structure. It is possible that this analysis could have been affected by biases imposed by the uneven sampling of *E. coli* targeted for genome sequencing, many of which originate from humans in countries such as the USA, Bangladesh or Tanzania (see Additional Table 1 for stain information). However, our analysis of the diversity within phylogroup A as represented by the sequenced population (see Additional Figure S2) indicates that, despite this uneven sampling, the structure of the phylogroup A phylogeny is incredibly well explored. Indeed, a newly sequenced phylogroup A *E. coli* can be expected to be, on average, as closely related to a previously sequenced genome as the evolutionary distance between some *E. coli* K-12 laboratory variants. This fine-grained representation of the phylogenetic diversity of phylogroup A in the sequenced population reduces the possibility of bias introduced by uneven sampling of *E. coli* from different environmental and geographical niches.

### MPEC are not more similar within-country than would be expected by chance

**Figure 3:**
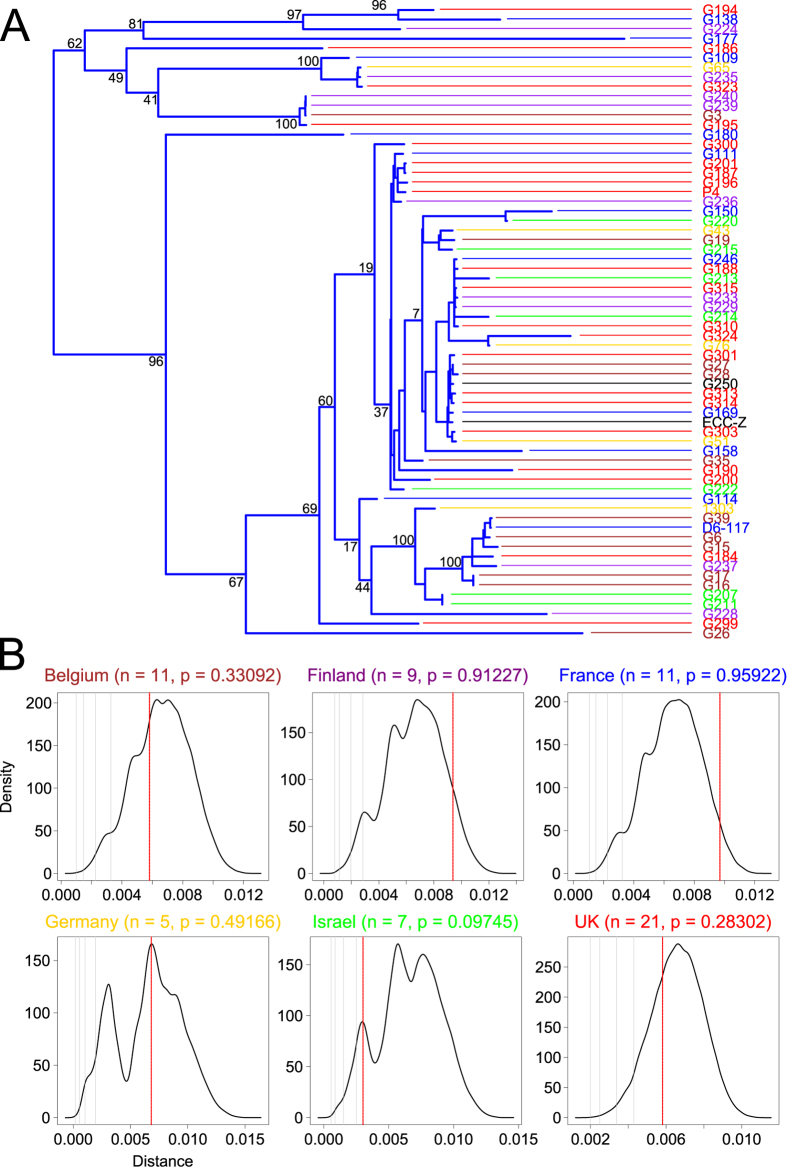
Within-country MPEC isolates are no more similar than would be expected by chance. Panel A shows a maximum likelihood tree of the 66 MPEC genomes used in this study, showing their relative positions within phylogroup A. Labels are coloured according to country of origin (Belgium = brown, Finland = purple, France = blue, Germany = gold, Israel = green, UK = red). One isolate (ECC-Z) was isolated from the Netherlands, and one from Denmark. Both these isolates are coloured black and due to the fact that they are the only representatives for their country groups these isolates were excluded from the analysis in this Figure. The countries of origin appear well mixed throughout the phylogenetic tree. Informative bootstrap values are given as integers adjacent to bifurcations. Panel B shows density estimates for the average phylogenetic distance observed between 10,000 randomised samples of the same number of genomes as isolates from each country (*n*, given alongside the country name for each plot), alongside a red vertical line which denotes the actual average distance between the *E. coli* genomes from each country. For each country, the average distance observed between the strains is no different than could be generated by a random process. The four grey vertical lines going right of the leading edge of the density plots represent p values of 0.0001, 0.001, 0.01, and 0.05, respectively.

The restriction of diversity exhibited by MPEC compared with other *E. coli* can be explained by two hypotheses. Firstly, it could be the case that this diversity limitation is a result of founder effects, whereby the only certain lineages of *E. coli* have had the opportunity to colonise the bovine udder and cause mastitis, and so these lineages are represented whilst others are not. Alternatively, it could be the case that the colonisation process is actively selective for particular *E. coli* strains, and promotes the proliferation only of particular lineages based on their inherent gene content. Our panel of phylogroup A MPEC originate from more than six different countries, and we reasoned that we could use this geographic distribution in order to test the hypothesis that opportunity (or founder effects) plays a role in limiting the molecular diversity of MPEC. To investigate this, we tested whether MPEC from one country tend to be more similar to each other than would be expected by chance since, if MPEC displayed significant within-country similarity, this may indicate that locally prevalent populations of MPEC have been founded by specific bacterial clones. To provide the data for this analysis, we examined a phylogenetic tree constructed from only the sixty-six MPEC genomes used in this study, and grouped the isolates according to their country of isolation (Fig. 3A). We then performed a similar analysis to that described for Fig. 2, and for each country group compared the average distances observed between these groups with a distribution of distances expected if these isolates were randomly positioned within the MPEC population (Fig. 3B).

These data show that, for the 6 countries investigated, MPEC are no more closely related within-country than would be expected from randomly sampling any MPEC, which indicates that, between-countries, the phylogenetic lineages of MPEC overlap. Although this comparison is not a direct test of the founder effect vs. selection hypotheses, we consider this data to contradict the founder effect hypothesis since, for this to be possible, the lack of observable within-country phylogenetic cohesion would necessitate that only the same lineages of *E. coli* in different countries have been given the specific opportunity to colonise the bovine udder and found MPEC communities - a scenario which is unlikely given the diversity of the phylogroup A population. Rather, it is more likely that the overlap in MPEC phylogeny is a result of a similar selective process operating in cattle in each country, which promotes the proliferation of similar lineages of MPEC, presumably based on their inherent gene content.

Together, the data summarised in Figs 2 and 3 support previous studies which have shown that the molecular diversity of MPEC may be lower than for other *E. coli*^10^,^22^, and suggests that not all *E. coli* are equally capable of causing mastitis. This hypothesis has some experimental support, since different *E. coli* strains vary in their ability to perform functions which may be important for mastitis, such as growth in milk, resistance to phagocytosis, or even fulfilling Koch’s postulates^10^,^21^,^30^. However, although those studies and our data suggest that founder effects are unlikely to play a major role in limiting the diversity of MPEC, further experiments are necessary to ensure that the observed inability for selected strains to cause bovine mastitis extends beyond a deficiency unique to *E. coli* K71^21^, for example.

### Mastitis-associated *E. coli* possess a larger core genome but a smaller pan-genome than is typical for phylogroup A

**Figure 4:**
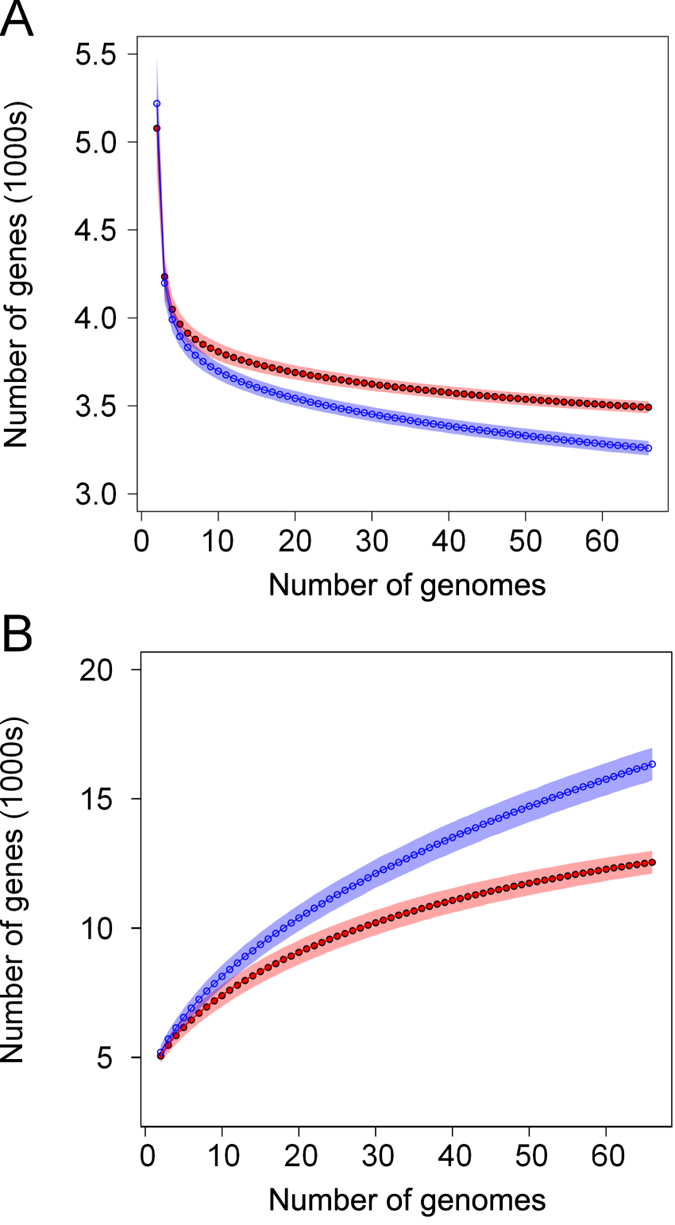
The core genome and pan genome size of the strains investigated. Panel A shows a curve for the core genome (genes present in at least *n*-1 strains) of phylogroup A *E. coli* (blue) and MPEC (red) when *n* number of strains are sampled from the populations, over 10,000 replications per data point. Polygons represent the standard deviation at each data point. This data shows that MPEC have a larger core genome than typical of phylogroup A. Panel B shows a curve for the pan-genome (genes present in at least one strain) for phylogroup A (blue) or MPEC (red) strains, when *n* number of genomes are sampled from the population, over 10,000 replications per data point. Polygons represent the standard deviation at each data point. These data shows that MPEC have a smaller pan-genome than phylogroup A *E. coli*.

Given that the molecular diversity of MPEC is significantly lower than would be expected from a random selection of phylogroup A isolates, next we investigated the gene content of these organisms to see if the restriction in phylogenetic diversity translated to a restriction of diversity at the gene content level. To do this, we estimated the pan-genome composition of the 533 phylogroup A *E. coli*, and compared the size of the core genome (genes present in all strains, Fig. 4A) or pan-genome (genes present in any strain, Fig. 4B) between MPEC and the general phylogroup A population. To calculate the curves shown in Fig. 4, we randomly sampled increasing numbers of genomes from both populations over 10,000 replications per data point, where the polygon surrounding the curve represents the standard deviation in the number of genes over the samples. For the analysis of core genes, and because many of the genome sequences used here are in draft form, we permit core genes to be absent in a maximum of one genome of the sample.

These data show clearly that the MPEC core genome is larger than expected for a similarly sized group of strains drawn at random from phylogroup A (Fig. 4A), while the MPEC pan-genome is much smaller than is typical for phylogroup A (Fig. 4B). On average, sixty-six phylogroup A strains encode a core genome of 3260 genes, whereas MPEC possesses a core genome of 3492 genes. Conversely, the pan-genome of 66 randomly drawn phylogroup A genomes averages at approximately 16349 genes, whereas MPEC lack thousands of genes otherwise found in phylogroup A, with a pan-genome of only 12558 genes.

The observation that the MPEC genome is concentrated with an expanded repertoire of core genes is consistent with our hypothesis that MPEC represents a specific pathotype or ecotype within the larger phylogroup A population and are more similar, both at the phylogenetic and gene content levels, than would be expected for a random selection of phylogroup A genomes. These data are suggestive of an active selection process operating in MPEC which purifies bacteria from the population when they lack necessary genes. These genes are then reflected in the specific MPEC core genome, ubiquitous in (and presumably necessary for) MPEC, yet presumably dispensable for the survival of other phylogroup A strains in other niches.

### Three loci form a specific phylogroup A MPEC core genome

For our analysis, we set out to detect genes which may be essential for the MPEC lifestyle, yet potentially dispensable for the survival of phylogroup A *E. coli* in their occupation of other environments. We reasoned that these genes would be represented by a subset of genes within the pan-genome that are found in the core genome of MPEC, yet are not found in the core genome of phylogroup A *E. coli* in general. To find these genes, first we modelled how the numerical abundance of genes in a population of 533 simulated genomes affected the probability that a gene would be captured in the core genome of sixty-six randomly sampled strains, over 100,000 replications. Since the data in Fig. 2 revealed that the chance of randomly selecting isolates as closely related to each other as MPEC are is 15 in 100,000, we used this as a threshold to determine genes that were statistically unlikely to be captured in the core genome of sixty-six sampled strains. The results of this modelling are shown in Additional Figure S3. This shows that a gene present in 446 or fewer genomes (in a population of 533 strains) can be expected to be captured in the core genome of sixty-six randomly sampled strains less than 15 in 100,000 times.

In light of this data, we probed the abundance of the genes in the pan-genome to identify those which were found in the core genome of MPEC, but no more than 446 of all phylogroup A genomes. This resulted in the identification of just nineteen genes, which we propose forms the MPEC-specifying core genome. These nineteen genes cluster into only three loci (Table 1).

The identification of nineteen genes clustering into just three loci instigated exploration of these genes. First we explored the distributions which causes these genes, some of which belong in operons alongside other genes, to be identified as MPEC core whilst their neighbours are not. In MG1655, *ymdE* is annotated as a pseudogene, and appears to be a 388 bp gene foreshortened by an IS3 element inserted on the reverse DNA strand. The *ymdE* in our pan-genome is the same length as that found in MG1655, indicating that we could not reliably detect a more complete representative of *ymdE* among sequenced phylogroup A genomes. In each case where we investigated the position of the coding sequence in the contigs of draft genomes, we found it to be encoded at a contig terminus, and any adjacent bases shared homology with the *insE1* gene found in MG1655. However, although several *E. coli* genomes from phylogroups other that phylogroup A encode a longer homologue of YmdE at 945 bp, in phylogroup A genomes, we could not detect any additional frameshift mutations, and only one genome had a nonsense mutation resulting in truncation of the coding sequence for *ymdE*. The lack of evidence for mutational attrition of *ymdE*, which may be expected to accrue if the pseudogene was selectively neutral, could suggest that this gene retains functionality. The *ymdE* gene contains domains consistent with acetyltransferase activity and, although it has not been characterised, there is evidence for *ymdE* transcription in the NCBI Gene Expression Omnibus (GEO) database.

**Figure 5:**
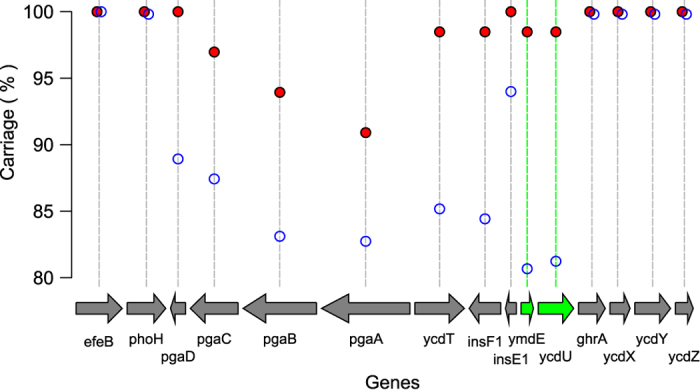
The carriage of the genes surrounding *ymdE* and *ycdU*. These data shows that several genes between *pgaD* and *ycdU* are more abundant in MPEC genomes from phylogroup A than they are in the wider phylogroup A population. These genes comprise what appears to be a genome island, flanked by the core genes *efeB/phoH*, and *ghrA/ycdXYZ*. Although *pgaB* and *pgaA*, along with *ymdE* and *ycdU*, are present in less than 446 of all phylogroup A (blue open circles), only *ymdE* and *ycdU* are also present in at least 65/66 MPEC genomes (red filled circles), qualifying these as MPEC-specific core.

The adjacent gene, *ycdU* was also identified by our analysis as part of the specific MPEC core genome. This gene is annotated in MG1655 as a putative membrane protein and has motifs associated with an inner membrane localisation including 8 transmembrane helices but, like *ymdE*, has not been functionally characterised. One MPEC genome encodes a truncated copy of *ycdU* near a contig boundary, but in this case we were able to identify the short remaining portion of the gene in another contig. Both *ymdE* and *ycdU* are encoded close to the biofilm-associated polysaccharide synthesis locus *pgaABCD*, and so we speculated that the presence of *ymdE* and *ycdU* in the specific MPEC core genome may serve as an indicator that these genes may also be commonly associated with MPEC. To test this, we extracted the nucleotide sequences of the genes surrounding *ymdE* and *ycdU* from MG1655, and profiled the distribution of these genes in phylogroup A and in MPEC (Fig. 5).

The data in Fig. 5 reveals some interesting aspects of the distribution of these genes in the *E. coli* phylogroup A population. Firstly, it is clear that a discrete region comprising the genes *pgaABCD, ycdT, insF1E1*, and *ymdE-ycdU*, is absent in at around ten to twenty percent of *E. coli* from phylogroup A. These are contrasted with the core flanking genes, *efeB*/*phoH*, and *ghrA/ycdXYZ*, found in almost all phylogroup A genomes. In this way, *pgaABCD, ycdT, insF1E1*, and *ymdE-ycdU* represent a region of genomic heterogeneity, with not all strains encoding these genes. Within this region of heterogeneity, and in addition to *ymdE-ycdU*, the *pgaABCD* genes also appear to be more common in MPEC than phylogroup A. However, the *pga* genes are clearly not ubiquitous in MPEC. Interestingly, in both phylogroup A and MPEC, there is trend for the *pga* genes to decrease in abundance compared with the downstream gene, so that whilst *pgaD* can be found in approximately 90% of all phylogroup A (100% of MPEC), *pgaA* is found only in approximately 80% of all phylogroup A (90% in MPEC). This suggests that the *pga* locus is relatively unstable and prone to attrition in different genomes of both MPEC and other phylogroup A strains. The *pga* genes are responsible for the biosynthesis of biofilm polysaccharides^31^, and so these data suggest against a consistent role for biofilms in mastitis.

Unlike this attrition of the *pga* genes, in MPEC, *ymdE* and *ycdU* are robustly co-maintained. The adjacent gene *ycdT* could also be considered a core MPEC gene, since it is present in 65/66 strains, however its abundance in other phylogroup A strains is sufficiently high that it can be captured in the core genome of sixty-six randomly sampled stains more than 0.015% of the time, so although we have not identified it as part of the specific MPEC core, both its distribution and proximity to *ymdE* and *ycdU* suggest that this gene could also contribute to the MPEC lifestyle. The *ycdT* gene is annotated in MG1655 as a membrane-anchored diguanylate cyclase. This type of gene regulates the turnover of the second messenger cyclic-di-GMP, which is known to affect behaviour such as motility, virulence, and biofilm production in a wide range of bacterial species^32^, and its homologue in *Yersinia* species (*hmsT*) regulates the *pga* genes (termed *hmsHFRS*) in that genus^33^. However, evidence suggests that although *ycdT* is co-regulated with the *pga* genes, the function of this gene is not linked to the expression of the *pga* operon^34^,^35^.

**Figure 6:**
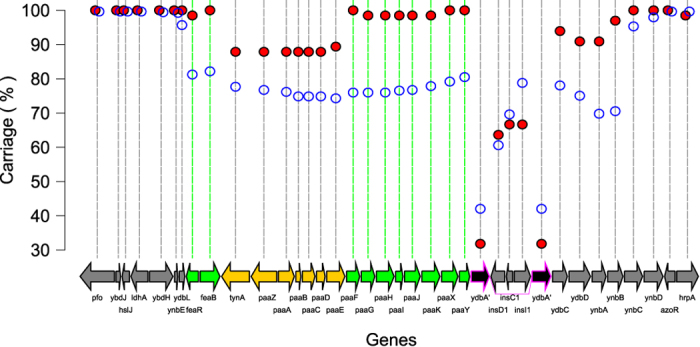
Carriage of the *paa* region in MPEC compared with phylogroup A *E. coli*. The core MPEC genes are coloured green, whilst a region of the *paa* locus which has been deleted in several MPEC is coloured yellow. The *ybdA* gene, which in MG1655 is a pseudogene, is outlined in magenta - the carriage for this gene is for the full length composite sequence from the MG1655 genome rather than for each half separately.

For the phenylacetic acid degradation locus which, in MG1655, consists of a seventeen gene locus encoded by *feaRB-tynA-paaZABCDEFGHIJKXY*^36^, we detected only ten of these (*feaR*, *feaB*, *paaFGHIJKXY*) to be components of the MPEC-specific core genome. We include the *feaR*, *feaB*, and *tynA* in the *paa* locus since these genes are involved in the metabolism of phenylethylamine to phenylacetic acid^37^,^38^, which provides substrate for the pathway encoded by the *paa* genes. We then analysed the distribution of the *paa* locus and surrounding genes in detail (Fig. 6).

Similar to the results for *ymdE* and *ycdU*, the locus comprising the *paa* genes appears as a region of genomic heterogeneity that occurs in the genome inserted between core genes such as *ydbH*, *ynbE*, and *ydbL* on one side, and *ynbC*, *ynbD*, and *ozoR* on the other. The region of heterogeneity comprising the *paa* genes also appears to include genes extending from *ydbA* (an autotransporter pseudogene in MG1655) to *ydbB*, which encode genes related to metabolism. Except for this autotransporter, for which full length versions occur in approximately 42% of all phylogroup A compared with 31% for MPEC, and the interrupting insertion sequences (*insD1*, *insC1*, *insI1*) which are found in MG1655, there is a clear differential in the carriage of the entire region in MPEC compared with all phylogroup A. However, in seven MPEC genomes a region of seven consecutive genes comprising *tynA-paaZABCDE* has been deleted (Additional Figure S4). These seven isolates (ECC-Z, G169, G250, G27, G301, G313, G314) are closely-related genetically, but were isolated in the UK, Denmark, Belgium and France, and one (ECC-Z) was isolated by another study in the Netherlands^39^ (see Fig. 1). One further, less closely related isolate (G246) possesses a similar deletion, although this genome contains *tynA* and *paaE*, but not *paaZABCD* (Additional Figure S4). One additional genome (G28) encodes *tynA*, but with only 78% identity to the sequence in MG1655.

These data indicate that the ability to catabolise phenylacetic acid could play a role in determining the fitness of MPEC. However, since strains in two independent lineages have accrued mutations in genes such as *paaZABCDE*, our data suggests that not all of the genes may be necessary to fulfil the functions required for this activity. When we examined which metabolic pathways these genes affected, we noticed that the *paa* genes which can be deleted in some MPEC mediate the conversion of phenylacetyl-CoA to 3-Oxo-5,6-dehydrosuberyl-CoA. The remaining *paa* genes (those which feature in the specific MPEC core genome) are involved in the upstream conversion of phenylacetaldehyde to phenylacetyl-CoA, and the downstream conversion of 3-Oxo-5,6-dehydrosuberyl-CoA to acetyl-CoA or succinyl-CoA both of which feed into the citrate cycle.

**Figure 7:**
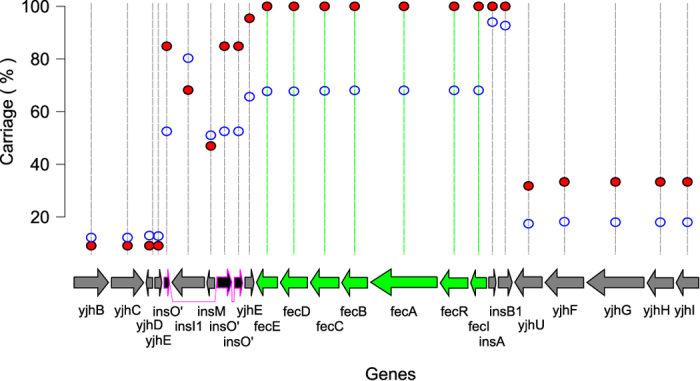
The carriage of the *fec* locus in MPEC and phylogroup A *E. coli*. This plot shows the genomic context of the *fecIRABCDE* genes in the genome of MG1655 and the percent carriage of each gene in the phylogroup A population (blue open circles) versus the MPEC population (red closed circles). The seven genes which form part of the specific MPEC core genome are coloured green. These genes (*fecIRABCDE*) confer the ability for the bacteria to utilise ferric citrate as a source of iron for growth. These genes are found in only 68% of all phylogroup A genomes, but are found in all of the 66 MPEC genomes we investigated. The genes flanking the *fec* locus show differing levels of carriage, which tend to be lower than that observed for the *fec* locus itself. This suggests that the genomic context of *fec* is different in different strains.

The final locus we identified as part of the specific MPEC core genome is the iron dicitrate utilisation pathway encoded by the *fecIRABCDE* genes. This locus shows a more simple distribution pattern than the mosaic observed for *paa* and is found in all MPEC strains, but only 68% of phylogroup A genomes (Fig. 7). Unlike the other specific MPEC core genes, the *fec* locus does not sit discretely in a region of heterogeneity flanked nearby with core genes. Instead, *fec* is encoded within a KpLE2 phage-like element which can be mobilised, even between different species of bacteria^40^. In MG1655 this region appears highly unstable, and we could ascribe nine insertion sequences in close proximity to the *fec* locus, as well as a number of pseudogenes. The *fec* genes are the most dramatically over-represented locus in the specific MPEC core genome.

Previous studies have hinted at the possible importance of ferric citrate utilisation in mastitis. Lin *et al*.^41^ showed that FecA production is prevalent in both *Klebsiella pneumoniae* and *E. coli* isolated from mastitis^41^, whilst Blum *et al*.^21^ recently showed that three MPEC strains encode *fec*, whilst a single isolate incapable of causing mastitis did not^21^. Citrate is a principal iron chelating agent in bovine milk^42^, and so it is likely that *fecIRABCDE* is required to efficiently sequester iron from milk to facilitate bacterial growth. Future experiments are planned to test the importance of Fec for MPEC growth in milk. It is also noteworthy that the citrate concentration of bovine milk is substantially higher than that reported in the milk of model animals such as mice and rats, which contain only trace levels of citrate in their milk^43^. This observation could have crucial implications for the use of mice and rats in models of mastitis infections.

Four MPEC isolates positioned within phylogroup A were not used in this study. These include two recently published genomes (VL2874 and VL2732)^21^ which were not available at the outset of our study, and two previously published genomes (ECA-O157 and ECA-727)^23^ which were excluded from our analysis due to a high number of contigs in the genome sequences for those strains. However, we have searched these four genomes and found that all nineteen core MPEC genes that we had identified in this study could be found in those strains (not shown) which is further confirmatory evidence that the genes we propose as MPEC core can be detected in other phylogroup A MPEC. In addition, to explore the possibility that these genes may represent general fitness determinants for bovine *E. coli* we queried the presence of these nineteen genes in our pan-genome data for the four sequenced genomes of *E. coli* known to be isolated from cattle (although not from mastitis, see Additional Table 1). We found that the *paa* genes are indeed common to all four bovine-associated strains, however, only *E. coli* DEC7B encodes the *fec* locus, and only *E. coli* CFSAN026796 and *E. coli* CFSAN026844 encode *ymdE* and *ycdU*. Unlike the MPEC genomes, no single strain of other bovine origin encodes all MPEC-specific core genes. A systematic study of bovine-associated *E. coli* is planned, however these data indicate that although the *paa* locus may be common to bovine strains, neither the *fec* locus nor *ymdE* or *ycdU* are core bovine-associated *E. coli* determinants.

## Conclusions

In this study we have examined the association between strains of *E. coli* from phylogroup A and mastitis at the population level. We focused our present analysis on phylogroup A for three principal reasons. Firstly, since phylogroup A are most often identified as amongst the most abundant phylogroup recovered from cases of bovine mastitis^10^,^11^,^12^,^13^,^14^, we wanted our study to have particular relevance to this predominant and problematic group. Secondly, since we speculated that selection plays a key role in determining which strains can or cannot elicit mastitis, we used phylogroup A as a discrete monophyletic group where pre-existing evidence pointed to the possibility that this group as a whole becomes enriched in mastitis vis-a-vis the external environment^10^. In this way, we identified lineages within phylogroup A that may be responsible for this enrichment. Thirdly, we were conscious of the fact that broad evolutionary distances (such as those which exist between the distinct phylogroups) may have profound effects on the distribution of genes, as a consequence of shared ancestry rather than functional relatedness. Such evolutionary distance may underpin some of the differences observed by others, since previous comparisons often involved few MPEC isolates versus comparator strains of disparate phylogenetic origins^21^,^23^,^26^.

We have found that phylogroup A MPEC (MPEC) tend to be more closely related to each other than would be expected if these bacteria had arisen at random within the population structure of phylogroup A, and have provided evidence which suggests that this is unlikely to be due to the fact that only a small number of lineages have had the opportunity to colonise the bovine udder (founder effects). Rather, our data suggests that an active selective process operates in mastitis, which permits the growth of certain strains whilst purifying others from this habitat. Our investigation of the pan-genome of phylogroup A and MPEC suggests that this selection operates at the level of just three key genetic loci that are ubiquitously present in MPEC but only sporadically present in the wider phylogroup A population. It is noteworthy that two of these three loci have metabolic functions whilst the third is of unknown function; an observation which further highlights the importance of anatomical niche nutritional milieu in pathogenicity, as shown functionally in an increasing number of studies with *E. coli* and related organisms. Notably, a recent population genomic study of *Klebsiella pneumoniae* also highlighted an association with metabolic loci among bovine mastitis strains although the specific metabolic determinants differed^44^.

The implications of these findings are that these genes and, hence, their products’ functions, may be essential for MPEC, yet dispensable for phylogroup A *E. coli* inhabiting other niches. Whilst less comprehensive, previous studies have hinted towards the involvement of ferric citrate uptake in mastitis pathogenicity, as well as the possibility that MPEC represent a specific pathotype or ecotype within *E. coli*, we consider this present work to provide the first substantive and statistically robust evidence that these bacteria contain of a core set of MPEC-specifying determinants which are actively selected for in the bovine udder, at least within phylogroup A *E. coli*.

Finally, it is important to note that the three genetic loci which we posit are crucial for mastitis in phylogroup A MPEC may not be the same for *E. coli* from other phylogroups. The products of genes which operate in a bacterium do so within a framework of the products of other genes co-resident in the genome, and these existing frameworks are likely to be more distinct the more distantly two *E. coli* are related. Characterisation of MPEC in other phylogroups will be carried out separately, and we postulate that a different subset of genes may be involved in mastitis in other phylogenetic backgrounds.

In sum, the analysis presented in this work provides strong evidence for candidate genes and functions involved in the successful colonisation and infection of the bovine udder by *E. coli* of phylogroup A and provides further evidence that adaptation to site-specifying nutritional milieu plays significant roles in niche-specific pathogenicity. We are actively perusing these candidates for further functional studies.

## Method

### Acquisition of published genome sequences

We downloaded the genome sequences for all 2951 *E. coli* available from public databases at the outset of the study. Since our planned analyses required good representation of the gene content for each isolate, we removed genomes where the number of contigs present in the assembly exceeded 400. This resulted in the exclusion of 202 genome sequences from further analysis. We found that two phylogroup A MPEC sequenced by a previous study (ECA-727 and ECA-O157)^23^ had contig counts which were higher than our thresholds, likely due to the low reported sequence coverage of these genomes^23^. As a result, these two genome sequences were excluded from further analysis. The 62 MPEC genomes newly sequenced in this study had contig counts of 100–350. Details of all strains used in the analysis are included in Additional Table 1.

### MPEC isolation and genome sequencing

Sixty-two MPEC were provided by the KOlimastIR consortium for genome sequencing. These strains were isolated from the milk of cows exhibiting clinical mastitis, using routine microbiological techniques, in diagnostic laboratories in origin countries. Total DNA was extracted using the Masterpure DNA Purification Kit (Epicentre, Madison, WI, USA) according to the manufacturer’s instructions. Sequencing was performed using an Illumina MiSeq sequencer at Glasgow Polyomics, Wolfson Wohl Cancer Research Centre, Glasgow, UK. A multiplex sequencing approach was used, involving 12 separately tagged libraries sequenced simultaneously in two lanes of an eight channel GAII flow cell. The standard Illumina Indexing protocol involved fragmentation of 2 μg genomic DNA by acoustic shearing to enrich for 200-bp fragments, A-tailing, adapter ligation and an overlap extension PCR using the Illumina 3 primer set to introduce specific tag sequences between the sequencing and flow cell binding sites of the Illumina adapter. DNA clean-up was carried out after each step to remove DNA < 150 bp using a 1:1 ratio of AMPure paramagnetic beads (Beckman Coulter, Inc., USA), and a qPCR was used for final DNA quantification. De novo genome assembly for each strain was carried out using CLC Genomics Workbench (version 6.5.2). Reads were trimmed by the removal of ambiguous nucleotides from read ends, and when quality scores fell below 0.001. Reads below 20 nucleotides were also removed. For assembly, default parameters were used (automatic bubble size, automatic word size), scaffolding was performed and paired distances were automatically detected. The minimum contig length was set to 200 bp. Genome sequences were uploaded to NCBI under the accession numbers given in Additional Table 1. The NCBI BioProject accession for this study is PRJNA305846.

### Elaboration of the *E. coli* population structure

To build an initial phylogenetic tree to confirm the placement of the 66 MPEC genomes into phylogroup A, we extracted the nucleotide sequences of 159 core genes from all of the *E. coli* genome sequences, aligned these genes by Muscle, concatenated them, and built a maximum likelihood tree under the GTR model using RaxML, as outlined previously^45^. Due to the size of this tree, bootstrapping was not carried out, although we have previously performed bootstrapping using these concatenated sequences on a subset of genomes which shows high support for the principal branches^45^.

### Phylogenetic estimation of phylogroup A *E. coli*

To produce a robust phylogeny for phylogroup A *E. coli* that could be used to interrogate the relatedness between MPEC and other *E. coli*, we queried our pan-genome data (see below for method) to identify 1000 random core genes from the 533 phylogroup A genomes, and aligned each of these sequences using Muscle. We then investigated the likelihood that recombination affected the phylogenetic signature in each of these genes using the Phi test^46^. Sequences which either showed significant evidence for recombination (p < 0.05), or were too short to be used in the Phi test, were excluded. This yielded 520 putatively non-recombining genes which were used for further analysis. These genes are listed by their MG1655 “b” number designations in Additional Table 2. The sequences for these 520 genes were concatenated for each strain. The Gblocks program was used to eliminate poorly aligned regions^47^, and the resulting 366312 bp alignment used to build a maximum likelihood tree based on the GTR substitution model using RaxML with 100 bootstrap replicates^45^.

### Phylogenetic tree visualisation and statistical analysis of molecular diversity

Phylogenetic trees estimated by RaxML were midpoint rooted using MEGA 5^48^ and saved as Newick format. Trees were imported into R^49^. The structure of the trees were explored using the ‘ade4’ package^50^, and visualised using the ‘ape’ package^51^. To produce a tree formed by only MPEC isolates, the phylogroup A tree was treated to removed non-MPEC genomes using the ‘drop.tip’ function within the ‘ape’ package- this tree was not calculated *de novo*.

To investigate molecular diversity of strains, branch lengths in the phylogenetic tree were converted into a distance matrix using the ‘cophenetic.phylo’ function within the ‘ape’ package, and the average distance between the target genomes (either all MPEC or country groups) was calculated and recorded. Over 100,000 replications, a random sample of the same number of target genomes were selected (66 for MPEC analysis, or the number of isolates from each country), and the average distance between these random genomes was calculated. The kernel density estimate for this distribution was then calculation using the ‘density’ function within R, and the actual distance observed for the target genomes compared with this distribution. To calculate the likelihood that the actual distance observed between the target genomes was generated by chance; the *p* value was calculated by the proportion of random distances which were as small, or smaller than, the actual distance. Significance was set at a threshold of 5%.

### Estimation of the phylogroup A pan-genome

To estimate the pan-genome of phylogroup A *E. coli*, we predicted the gene content for each of the 533 genomes using Prodigal^52^. We initially attempted to elaborate the pan-genome using an all-versus-all approach used by other studies and programs^53^,^54^,^55^,^56^,^57^,^58^, however the number of genomes used in our analysis proved prohibitive for the computing resources available. Instead, we adapted the iterative approach used by Holt *et al*.^59^. In our implementation, the pan-genome was initiated as the nucleotide sequences predicted for the genes of the first genome used (the input order of genomes was randomised). The nucleotide sequences of the genes for the genome in the next iteration (*Gi*) was then compared with the pan-genome using MUMmer (Nucmer algorithm, parameters used were: -forward −l 20 −mincluster 20 −b 200 -maxmatch)^60^. The results of the MUMmer analyses were parsed to capture gene pairs which shared greater than 95% homology. Homology was calculated as the average of percent sequence identity, the percent coverage of the query sequence by the reference, and the percent coverage of the reference sequence by the query. This list of nodes (genes) and edges (homology) was then used as input data for the graph building algorithm, MCL^61^. The resulting graphs were explored to identify genes in *Gi* which shared a graph with genes already present in the pan-genome - these genes were excluded, however the number of times a gene was matched to the existing pan-genome was found in additional genomes was recorded. All genes not sharing graphs with genes already present in the pan-genome were added to the pan-genome for use in the next iteration. After each genome had been compared with the pan-genome, we performed an amalgamation step to attempt to detect genes which, in draft genomes, had been split over multiple contigs. To do this, we compared the pan-genome against itself using MUMmer under the same parameters as previously specified. In this case, however, we recorded gene pairs when the following criteria were met: i) the length of the query sequence was less than 80% of the length of the reference sequence, ii) the length of the reference sequence was greater than 120% the length of the query sequence, iii) the alignment identity was greater than 95%, iv) the coverage of the reference by the query sequence was greater than 20%, and v) the coverage of the reference by the query sequence was less than 80%. When these criteria were met, we defined the query sequence as ‘part-of’ the reference. These pairs were then passed to MCL for graph building. For each graph, the longest gene which could be detected in three or more individual genomes was captured as the representative gene for the graph, all other genes were discarded. This step was designed to detect the longest representative of a set of gene parts when that representative could be reliably detected. This detection threshold of three separate genomes was selected in order to limit the possibility that gene fusions created by sequencing error (which may be expected to be very rare within the genes of each graph) would be chosen to replace ‘true’ genes, whilst allowing full length representatives of genes split over contigs (which may be expected to be more common, since at least some of the genomes within our sample originate from completely sequenced isolates) to be recovered. Finally, the repaired genes in the pan-genome were again compared against themselves using MUMmer, under the same parameters as before. This time, gene pairs were assigned when two genes shared greater than 80% homology (homology was again defined as the average of percent identity, percent coverage of the reference by the query, and percent coverage of the query by the reference). These pairs were passed to MCL for a final round of graph building, and a single representative for each graph (which represents gene families) was saved. This final step, where gene families sharing 95% homology are condensed to gene families sharing 80% homology was necessary to address the problem presented by triangle inequality. For example, if the iterative approach is used to capture gene families which share greater than 80% homology without this final step, the input order of genomes will profoundly affect the final number of genes estimated in the pan genome. Consider the following simplified three gene scenario using a similarity threshold of 80%: Gene A matches gene B and gene C at 81% identity, although genes B and C match each other at 79% identity. If gene A is encountered in the first iteration, it can be compared to either genes B or C next, and finally retained as the sole representative of this gene family in the pan-genome (even though genes B and C only match each other to 79%, since in this scenario genes B and C are never directly compared). However, if gene B is encountered first, it can be compared to gene A, where gene B will then be retained in the pan-genome. Then, in the next iteration where genes B and C are compared, both these genes are retained in the pan-genome since they match with an identity 1% below the required threshold. This hypothetical scenario (but drawn from problems we encountered) represents a discretisation problem which is difficult to resolve without an all-versus-all approach, which is provided for by the final step - the purpose of the iterative steps is to broadly capture genes which share greater than 95% homology in order to limit the number of genes used in the final all-versus-all comparison. At each stage, the genomes in which these genes could be detected was tracked, which allowed the data to finally be transformed into a binary presence/absence matrix for further investigation.

To investigate the size of the core or pan-genomes of phylogroup A or MPEC strains, for each data point we randomly sampled (with replacement) *n* number of strains from our pan-genome presence absence matrix data for 10,000 replications, where *n* is an integer between 2 and 66. For the core genome, for each data point a gene was counted as ‘core’ if it was present in *n*-1 genomes. For the pan genome, a gene was counted if it was present in at least one genome.

### Determination of the specific MPEC core genome

To determine the genes that could be detected in all MPEC (core genes), but which were not represented in the core genome of a similarly sized sample of all phylogroup A genomes, first we modelled how the numerical abundance of a gene in the phylogroup A population affected the probability that this gene would be captured in the core genome of 66 sampled strains. To do this, we simulated random distributions of increasing numbers of homologues (from 1 to 533) in 533 genomes over 100,000 replications per data point. For each replication, we sampled 66 random genomes and counted how many times a gene with that numerical abundance in 533 genomes appeared in at least 65 of the 66 sampled genomes. We then fit a curve to this data using the ‘lm’ function within R using the third degree polynomial. Since our data intimated that randomly sampled *E. coli* could be expected to be as closely related to each other as MPEC are 15 in 100,000 times, we set the lower limit of the number of times a homologue could be detected in at least 65/66 sampled strains to be considered ‘core’, also, as 15 in 100,000. By extrapolating from the fitted curve, we found that if a homologue was present in more than 446/533 genomes, that homologue could be expected to be captured in at least 65/66 strains in greater than 15 in 100,000 times. In this way, we defined specific MPEC core genes as those present in at least 65 of 66 MPEC genomes, but 446 or fewer of the 533 phylogroup A genomes.

### Further examination of the specific MPEC core genes

To further investigate the 19 genes which formed the specific MPEC core genome, we took the nucleotide sequences for these genes from our pan-genome and compared these, using BLAST, to the genome sequence for MG1655 (accession U00096). It should be noted that one gene was a fusion of *paaA* and *paaK*. This results from the observation that several genomes, including DEC6A, DEC6B amongst others, appear to contain a deletion in several *paa* genes which has resulted in the fusion of *paaA* and *paaK* being represented in our pan-genome. Further investigation showed that *paaK*, but not *paaA*, to be in the specific MPEC core genome, since *paaA* is part of the *paa* locus deleted in some MPECs (a separate deletion to the event which has resulted in the fusion of *paaA* and *paaK*). Except for this anomaly, all 19 MPEC core genes were found in the MG1655 genome with greater than 95% identity, and so we assumed the annotation from the MG1655 genome onto our set of 19 genes.

To confirm the distribution of genes in our pan genome and to further investigate the distribution of nearby genes, we extracted the nucleotide sequences of genes from the MG1655 genome using the Artemis genome browser^62^, and probed for the presence of these genes in the 533 phylogroup A genomes using BLAST. Presence of a gene was ascribed by sequences within the target genome sharing greater than 80% identity with the gene sequence from MG1655.

### Figure generation and formatting

All Figures were produced using R and associated packages, and formatted using Inkscape version 0.48 supplemented with the Ghostscript 9.14 extension for the manipulation of encapsulated postscript (eps) files. Figures were manipulated for scale, labelling and colouring without affecting the representation of data.

## Additional Information

**Accession codes:** The Whole Genome Shotgun sequences from this project have been deposited at DDBJ/ENA/GenBank under the accessions given in Table S1.

**How to cite this article**: Goldstone, R. J. *et al*. Genomic content typifying a prevalent clade of bovine mastitis-associated *Escherichia coli*. *Sci. Rep*. **6**, 30115; doi: 10.1038/srep30115 (2016).

## Supplementary Material

Supplementary Information

## Figures and Tables

**Table 1:** Nineteen genes form the specific phylogroup A MPEC core genome.

Gene in MG1655	Gene name	Product	% in MPEC	% in phy A	cluster
b1028	*ymdE*	undefined product	98.5	79.9	1
b1029	*ycdU*	putative inner membrane protein	98.5	82.0	1
b1384	*feaR*	transcriptional activator for *tynA* and *feaB*	100	82.1	2
b1385	*feaB*	phenylacetaldehyde dehydrogenase	100	82.2	2
b1393	*paaF*	2,3-dehydroadipyl-CoA hydratase	100	79.2	2
b1394	*paaG*	1,2-epoxyphenylacetyl-CoA isomerase, oxepin-CoA-forming	100	76.7	2
b1395	*paaH*	3-hydroxyadipyl-CoA dehydrogenase, NAD + -dependent	98.5	76.0	2
b1396	*paaI*	hydroxyphenylacetyl-CoA thioesterase	98.5	76.6	2
b1397	*paaJ*	3-oxoadipyl-CoA/3-oxo-5,6-dehydrosuberyl-CoA thiolase	98.5	76.6	2
b1398	*paaK*	phenylacetyl-CoA ligase	98.5	76.7	2
b1399	*paaX*	transcriptional repressor of phenylacetic acid degradation *paa* operon, phenylacetyl-CoA inducer	100	79.2	2
b1400	*paaY*	thioesterase required for phenylacetic acid degradation; trimeric; phenylacetate regulatory and detoxification protein; hexapeptide repeat protein	100	80.5	2
b4287	*fecE*	ferric citrate ABC transporter ATPase	98.5	66.6	3
b4288	*fecD*	ferric citrate ABC transporter permease	100	67.9	3
b4289	*fecC*	ferric citrate ABC transporter permease	100	67.9	3
b4290	*fecB*	ferric citrate ABC transporter periplasmic binding protein	100	68.1	3
b4291	*fecA*	TonB-dependent outer membrane ferric citrate transporter and signal transducer; ferric citrate extracelluar receptor; FecR-interacting protein	100	68.1	3
b4292	*fecR*	anti-sigma transmembrane signal transducer for ferric citrate transport; periplasmic FecA-bound ferric citrate sensor and cytoplasmic FecI ECF sigma factor activator	100	68.1	3
b4293	*fecI*	RNA polymerase sigma-19 factor, fec operon-specific; ECF sigma factor	100	68.1	3

These genes cluster into three loci, including two adjacent genes *ymdE* and *ycdU*, ten genes from the phenylacetic acid degradation operon (*feaR*, *feaB*, *paaFGHIJKXY*) and the seven genes of the ferric citrate uptake system (*fecIRABCDE*).
