# Publisher Correction: Genomic content typifying a prevalent clade of bovine mastitis-associated *Escherichia coli*

**DOI:** 10.1038/s41598-020-72806-w

**Published:** 2020-09-25

**Authors:** Robert J. Goldstone, Susan Harris, David G. E. Smith

**Affiliations:** Heriot-Watt University, School of Life Sciences, Edinburgh Campus, EH14 4AS Scotland, UK

Correction to: *Scientific Reports* https://doi.org/10.1038/srep30115, published online 20 July 2016

A supplementary file containing Additional Table 1 is missing from this Article and is provided below.

## Supplementary information

Additional Table 1.

