# Supplementary material for: Genomic content typifying a prevalent clade of bovine mastitis-associated Escherichia coli
Source: Sci Rep. 2016 Jul 20;6:30115. doi: 10.1038/srep30115 (PMC4951805; doi:10.1038/srep30115)
Supplement: Supplementary Information [file srep30115-s1.pdf]

Genomic content typifying a prevalent clade of bovine mastitis-associated *Escherichia coli*:

Additional information

Robert J. Goldstone[1]

Susan Harris[1]

David G. E. Smith[1,*]

[1] Heriot-Watt University, School of Life Sciences, Edinburgh Campus, EH14 4AS

*Corresponding author

Additional figure S1

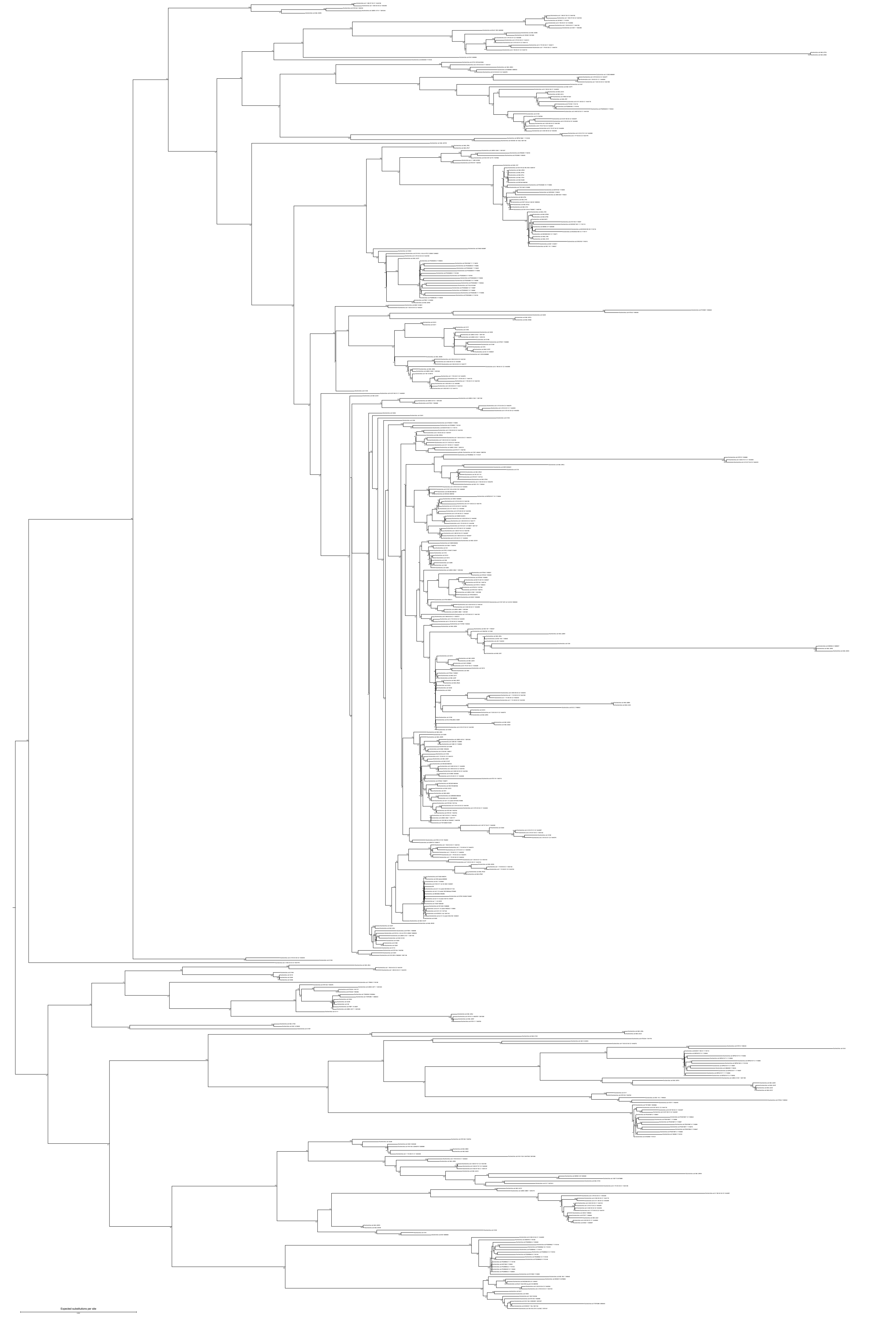

Figure S1. Maximum likelihood phylogenetic tree for 533 phylogroup A *E. coli* genome sequences constructed using the concatenated sequences for 520 core genes. Bootstrap values (100 replicates) are shown.

Additional figure S2:

Does the uneven representation of *E. coli* from different countries or ecological niches in the sequenced *E. coli* phylogroup A strains compromise the representation of the diversity in the sequenced population?

Despite the possible biases in the sampling of *E. coli* which have been used for genome sequencing, many of which come from humans, including a large cohort of sequences from Bangladesh, the USA or Tanzania, we observe that the phylogenetic diversity of phylogroup A is well saturated by the whole sample and that neither country nor host origin bias representativeness.

To demonstrate this we performed an analysis in which, by comparing a single query genome against numerically increasing random samples of the population (over 10,000 replications per data point), we ask how similar the query strain is to its most similar neighbour in the sample. This analysis provides a description of how similar a newly sequenced genome can be expected to be to one already present in the sequenced population, depending on the size of the sequenced population. Figure S2 shows that as individuals are added to the sequenced population the average minimum distance observed between a query genome and a genome already present in the sequenced population rapidly approaches zero. In fact, at the end of the sampling the average minimum distance observed between a new and a previously seen strain (d =~ 0.0005) is broadly equivalent to the evolutionary distance between several of the *E. coli* K-12 laboratory variants, such as DH5-α and S17 (d = 0.0005). These data indicate that a newly sequenced phylogroup A *E. coli* can be expected to be, on average, as similar to at least one previously sequenced isolate as the evolutionary distance between K-12 variants, which are of negligible evolutionary distance. This would suggest that the phylogeny of phylogroup A *E. coli* is incredibly well explored and substantially

reduces the possibility of any bias introduced by uneven representation of *E. coli* from different environmental and geographical niches.

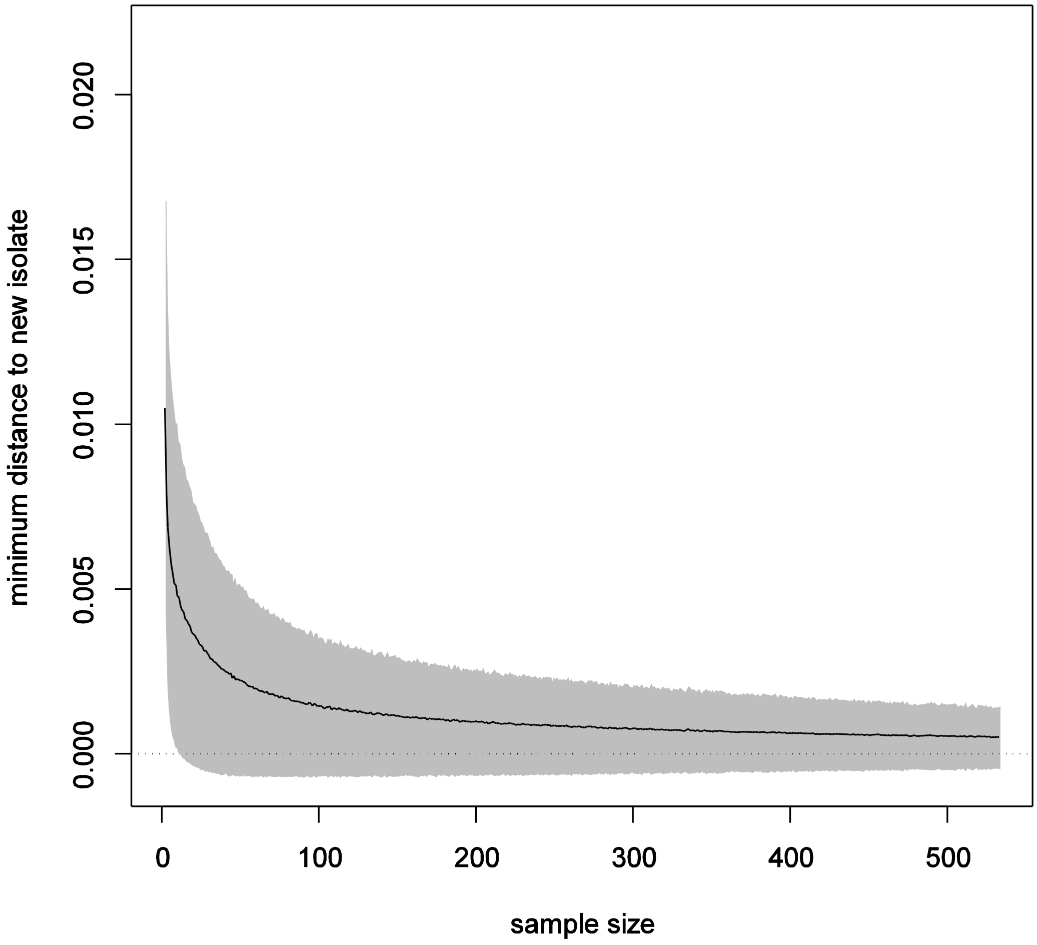

Figure S2. The distance between a new genome and those present in a sequenced population of increasing sizes. These data show that as the size of the previously sequenced population (sample size) increases, the distance (solid black curve) found between this strain and its nearest neighbour in the sequenced population decreases rapidly. The grey polygon surrounding the curve represents the standard deviation of distances, based on 10,000 replications per data point. These data indicate that the overall phylogenetic representation of phylogroup A is not compromised by biases inherent in the sampling of global *E. coli* which have been sequenced.

Additional Figure S2. Modelling the interaction between the numerical abundance of a gene and the likelihood that gene will be captured in the core genome of sampled strains

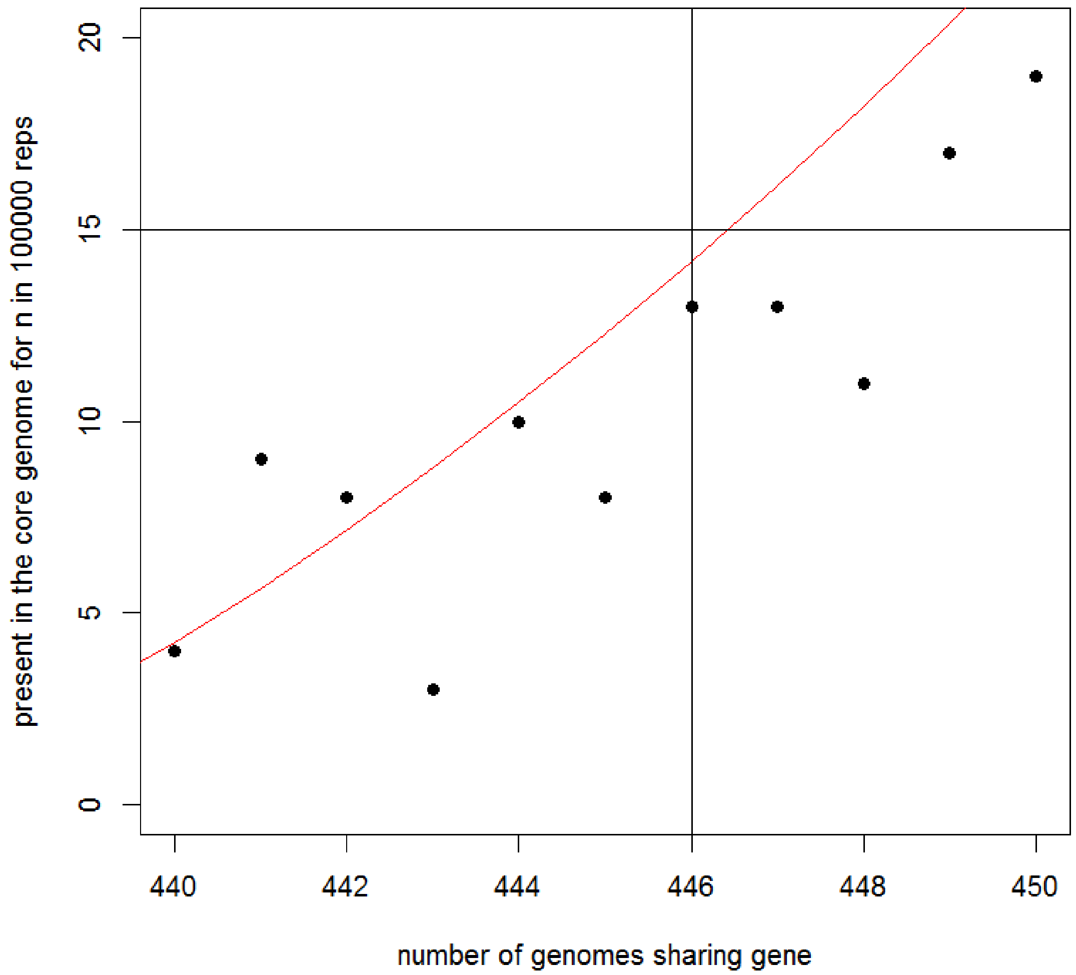

Figure S2. The interaction between the abundance of a gene in a population of 533 genomes and the number of times that gene is found in at least 65/ 66 sampled strains. To identify the genes which comprise the specific PAMPEC core genome, first we wanted to investigate how numerically abundant a gene needed to be in a population of 533 strains to be captured in the core genome of 66 randomly sampled genomes captured we modelled how the numerical abundance of a gene in a population of 533 *E. coli* affected the number of times that gene could be expected to be captured in the core genome of 100,000 random samples of 66 strains. To do this, we simulated random

distributions of increasing numbers of homologues (from 1 to 533) in 533 genomes over 100,000 replications per data point. For each replication, we sampled 66 random genomes and counted how many times a gene with that numerical abundance in 533 genomes appeared in at least 65 of the 66 sampled genomes. We then fit a curve to this data using the 'lm' function within R using the third degree polynomial. Since our data intimated that randomly sampled *E. coli* could be expected to be as closely related to each other as MPEC are 15 in 100,000 times, we set the lower limit of the number of times a homologue could be detected in at least 65 / 66 sampled strains to be considered 'core', also, as 15 in 100,000. By extrapolating from the fitted curve, we found that if a homologue was present in more than 446 / 533 genomes, that homologue could be expected to be captured in at least 65 / 66 strains greater than 15 in 100,000 times. In this way, we defined specific PAMPEC core genes as those present in at least 65 of 66 PAMPEC genomes, but 446 or fewer of the 533 phylogroup A genomes.

Additional figure S3. Deletion of regions of the *paa* locus in some MPECs

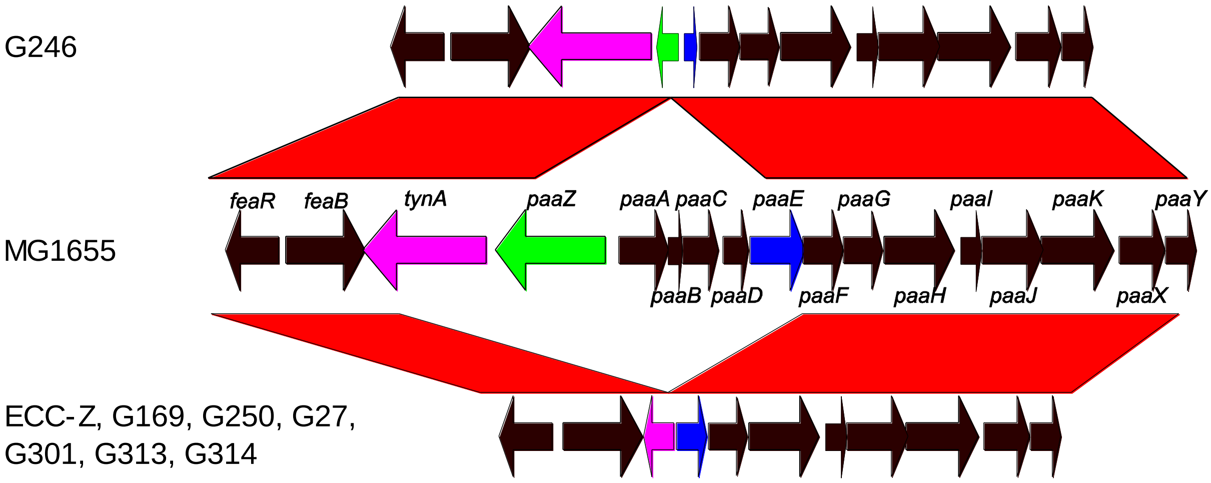

Figure S3. Deletion of some genes in the *paa* locus in a subset of MPEC. By comparing the *paa* locus from MG1655 to several MPEC genomes we identified 7 genomes which had a deletion in the region comprising *tynA-paaZAECDE,* and one further genome with a separate deletion in *paaZABCDE*.

Additional table 2. A list of genes used for the phylogenetic analysis of phylogroup A genomes. This list gives the “b” designations from the MG1655 genome (uid:U00096).

b0006	b0391	b0707	b0881	b1179	b1709
b0009	b0393	b0721	b0891	b1184	b1710
b0010	b0394	b0722	b0893	b1187	b1723
b0014	b0399	b0728	b0896	b1194	b1725
b0015	b0401	b0734	b0898	b1207	b1728
b0050	b0404	b0735	b0907	b1208	b1739
b0055	b0405	b0738	b0911	b1209	b1749
b0058	b0409	b0750	b0920	b1213	b1765
b0080	b0411	b0751	b0921	b1219	b1768
b0083	b0413	b0753	b0922	b1232	b1778
b0087	b0421	b0754	b0923	b1233	b1782
b0089	b0422	b0755	b0945	b1236	b1784
b0091	b0424	b0782	b0958	b1242	b1795
b0094	b0430	b0785	b0960	b1243	b1802
b0103	b0432	b0791	b0962	b1245	b1806
b0104	b0436	b0795	b0963	b1246	b1807
b0108	b0443	b0796	b0964	b1253	b1808
b0110	b0446	b0798	b0965	b1254	b1825
b0129	b0450	b0802	b0966	b1255	b1840
b0147	b0453	b0804	b0970	b1275	b1841
b0154	b0454	b0807	b1000	b1282	b1846
b0157	b0463	b0810	b1009	b1293	b1848
b0158	b0472	b0811	b1010	b1299	b1850
b0166	b0475	b0812	b1013	b1429	b1856
b0169	b0487	b0814	b1048	b1452	b1857
b0170	b0490	b0815	b1054	b1521	b1861
b0171	b0528	b0817	b1056	b1531	b1865
b0172	b0605	b0824	b1061	b1608	b1874
b0173	b0607	b0825	b1064	b1609	b1905
b0174	b0611	b0828	b1066	b1635	b2101
b0176	b0614	b0832	b1069	b1636	b2134
b0178	b0618	b0835	b1103	b1637	b2137
b0179	b0620	b0836	b1104	b1638	b2150
b0187	b0623	b0838	b1105	b1640	b2151
b0196	b0624	b0841	b1106	b1641	b2153
b0200	b0628	b0843	b1110	b1643	b2158
b0213	b0630	b0853	b1116	b1651	b2179
b0215	b0631	b0858	b1120	b1655	b2181
b0222	b0638	b0862	b1132	b1656	b2187
b0384	b0658	b0864	b1133	b1662	b2197
b0386	b0659	b0865	b1134	b1676	b2203
b0388	b0683	b0876	b1174	b1703	b2204

b2205	b2617	b3151	b3348	b3705	b3960
b2218	b2618	b3152	b3351	b3725	b3964
b2236	b2668	b3153	b3354	b3735	b3973
b2237	b2669	b3154	b3357	b3738	b3974
b2241	b2671	b3163	b3364	b3742	b4012
b2250	b2682	b3164	b3386	b3764	b4023
b2267	b2684	b3165	b3389	b3780	b4046
b2280	b2689	b3167	b3391	b3784	b4056
b2282	b2702	b3175	b3394	b3786	b4059
b2285	b2748	b3177	b3395	b3790	b4062
b2287	b2749	b3188	b3397	b3791	b4063
b2288	b2750	b3189	b3399	b3793	b4135
b2290	b2780	b3191	b3405	b3806	b4136
b2295	b2782	b3192	b3414	b3810	b4141
b2296	b2793	b3194	b3415	b3811	b4147
b2303	b2809	b3195	b3416	b3812	b4149
b2311	b2811	b3198	b3417	b3824	b4154
b2313	b2812	b3199	b3424	b3825	b4160
b2316	b2818	b3204	b3437	b3827	b4161
b2319	b2825	b3205	b3453	b3832	b4162
b2323	b2828	b3206	b3460	b3836	b4171
b2398	b2829	b3228	b3461	b3838	b4178
b2416	b2832	b3230	b3466	b3842	b4188
b2432	b2837	b3231	b3467	b3848	b4201
b2436	b2891	b3233	b3471	b3850	b4203
b2477	b2895	b3235	b3472	b3869	b4214
b2478	b2897	b3236	b3473	b3871	b4232
b2513	b2899	b3238	b3475	b3887	b4236
b2525	b2900	b3239	b3478	b3892	b4237
b2527	b2927	b3248	b3499	b3919	b4259
b2528	b2936	b3249	b3503	b3921	b4362
b2529	b2946	b3250	b3560	b3922	b4363
b2533	b2951	b3251	b3570	b3924	b4372
b2540	b2990	b3255	b3592	b3926	b4395
b2552	b2992	b3257	b3602	b3927	b4397
b2553	b2993	b3260	b3611	b3930	b4398
b2559	b3005	b3279	b3634	b3931	b4461
b2565	b3011	b3282	b3635	b3932	b4554
b2568	b3032	b3283	b3643	b3933	
b2570	b3034	b3295	b3646	b3934	
b2571	b3058	b3303	b3648	b3936	
b2573	b3059	b3314	b3649	b3937	
b2587	b3068	b3321	b3654	b3941	
b2595	b3071	b3340	b3666	b3945	
b2597	b3148	b3341	b3668	b3958	
b2608	b3149	b3342	b3697	b3959	
